# A Single Variant in Pri-miRNA-155 Associated with Susceptibility to Hereditary Breast Cancer Promotes Aggressiveness in Breast Cancer Cells

**DOI:** 10.3390/ijms232315418

**Published:** 2022-12-06

**Authors:** Natalia Landeros, Patricio Gonzalez-Hormazabal, Pablo Pérez-Moreno, Julio C. Tapia, Lilian Jara

**Affiliations:** 1 Programa de Genética Humana, Instituto de Ciencias Biomédicas, Facultad de Medicina, Universidad de Chile, Santiago 8380453, Chile; 2 Programa de Biología Celular y Molecular, Instituto de Ciencias Biomédicas, Facultad de Medicina, Universidad de Chile, Santiago 8380453, Chile

**Keywords:** microRNA, breast cancer, SNV, canonical Wnt pathway, aggressiveness

## Abstract

Variants in genes encoding for microRNAs have been associated with their deregulation in breast cancer (BC). Sequencing of microRNAs deregulated in BC was performed using DNA from Chilean patients with a strong family history and negative for mutations in BRCA1/BRCA2. Seventeen variants were identified, three of which were selected for a case-control association study: rs376491654 (miR-335), rs755634302 (miR-497), and rs190708267 (miR-155). For rs190708267 C>T, the heterozygous T allele was detected in four BC cases and absent in controls, while homozygous TT cases were not detected. Variants were modelled in silico, cloned in a plasmid, expressed in BC cell lines, and functional in vitro assays were performed. Overexpression of the miR-155-T allele increased mature miR-155-5p levels in both BC cell lines, suggesting that its presence alters pre-miR-155 processing. Moreover, BC cells overexpressing the miR-155-T allele showed increased proliferation, migration, and resistance to cisplatin-induced death compared to miR-155-C overexpressing cells. Of note, the 3′UTR of APC, GSK3β, and PPP1CA genes, all into the canonical Wnt signaling pathway, were identified as direct targets. APC and GSK3β mRNA levels decreased while PP1 levels increased. These results suggest a pathogenic role of the variant rs190708267 (miR-155) in BRCA 1/2 negative BC, conferring susceptibility and promoting traits of aggressiveness.

## 1. Introduction

Breast cancer (BC) is the most diagnosed cancer and the leading cause of cancer death in women worldwide [1]. In Chile, the mortality rate is 15.8/100,000 women [2]. Most cases are considered sporadic, but there is a 20% that has an identified genetic component or hereditary predisposition to develop BC. On half of them, the genetic etiology has not been identified yet, and these families are called BRCA 1/2 negative [3]. According to the BC polygenic model, an unfavorable combination of genetic variants in moderate and low penetrance genes could be responsible for a significant number of cases of familial BRCA 1/2 negative BC [4].

Initially, the search for new predisposing genes has been focused mainly on those that encode proteins. Nevertheless, it has been switched to non-coding genes, in particular microRNAs (miRNAs or miRs). Iorio et al., 2005 [5], published one of the first investigations that reported the presence of a pattern of deregulated miRNAs in BC. They compared the expression levels of miRNAs in 76 samples of BC tumors and 10 healthy breast tissues by microarray and identified 29 differentially expressed miRNAs in BC. Among the most deregulated miRNAs were miR-10b, miR-125b, miR-145, miR-21, and miR-155, suggesting that they could be used as diagnostic or prognostic molecular biomarkers [5].

Subsequent studies revealed a profile of miRNAs commonly deregulated in BC, with an increase or decrease of a specific group of them [6,7,8,9]. These alterations in the expression pattern of miRNAs may promote certain aggressiveness traits, including increase in proliferation, death resistance, invasion, metastasis, and drug resistance [9,10]. One of the miRNAs commonly deregulated in BC is miR-155 [11,12], which is transcribed from the *MIR155HG* gene located in chromosome 21q21.3. Under physiological conditions, miR-155 plays an essential role in hematopoiesis, inflammation, and immune response [13,14,15]. In pathological conditions, miR-155 levels are elevated, and several studies have correlated its overexpression with progression and pathogenesis in many cancers, including BC, suggesting that this miRNA leads to tumorigenesis and increased malignancy. This effect is because miR-155 has a large number of mRNA targets involved in different cellular processes associated with cancer, such as apoptosis, tumor growth, epithelial-mesenchymal transition (EMT), invasion, and metastasis, among others [16,17,18,19].

The single nucleotide variants (SNVs) present in miRNA genes could explain the aberrant expression of some of them. Depending on the SNV location, it may alter transcription or processing of pri-miR or pre-miR by increasing or decreasing the levels of the mature form. Besides, if the SNV is located in the mature miRNA, it may alter its biological function by modifying its ability to bind its mRNA target [20,21,22,23]. In this work, we aimed to identify SNVs present in miRNA genes of a Chilean population of BRCA 1/2 negative BC patients. In order to establish their putative contribution to BC aggressiveness, the functional effects of some SNVs were studied in BC cell lines. The findings obtained in this work allow us to suggest a pathogenic role of the variant rs190708267 (miR-155-5p) by both conferring susceptibility and promoting traits of aggressiveness to BRCA 1/2 negative BC patients.

## 2. Results

### 2.1. Variant Identification in miRNA Genes of BC Patients

Eleven miRNAs (miR-10b, miR-21, miR-125a, miR-145, miR-155, miR-497, miR-195, miR-221, miR-222, miR-335, and miR-520c) were selected following the criteria described in Methodology. Those miRNAs have been reported to be deregulated in BC and are associated with carcinogenesis, progression, and metastasis. To identify variants that could explain this deregulation, we sequenced these miRNAs in 100 Chilean probands from BC families negative for *BRCA1* and *BRCA2* point mutations, which had three or more BC cases in a single family. The average age of diagnosis was 45 years (range 21 to 74 years). In addition, 24% of these cases had a history of bilateral BC and 14% of these cases had a history of ovarian cancer (Table S1).

We identified 17 SNVs in eight out of the eleven sequenced microRNAs (Table 1). No variants were detected in three miRNAs: miR-145, miR-222, and miR-520c. From the identified variants, three correspond to insertions and fourteen to substitutions. Two of the identified substitutions were not described previously in the literature or in the dbSNP database. Each was detected in one out of the 100 sequenced probands, respectively. These new SNVs are located at the 3′ end of the pri-miR-10b sequence. One variant corresponds to n.300 G>T, which is 189 bp from the pre-miRNA end, and the other one is an n.310 C>T substitution, located at 199 bp of the pre-miRNA end. Both variants were found in 1% of sequenced patients, suggesting that are new variants for the Chilean population. In the Ensembl database (ENSG00000207744), it was reported that the *miR-10b* gene is within an intron of the *HODX3* (Homeobox D3) gene. However, since they are also located quite far from the donor and acceptor sites of splicing, they would not interfere with the maturation of the *HOXD3* mRNA. Thus, both variants would not have a greater effect on the protein.

### 2.2. Association Study of Variants rs376491654, rs755634302, and rs190708267

From the 17 identified variants, those that accomplish the following criteria were selected for the association study: (i) variants located in/or near (max 50 bp) the pre-miRNA sequence; (ii) variants whose allelic frequency (determined in the 100 patients) was higher than the one registered in the dbSNPs database for the general population (Table S2); and (iii) variants that alter the secondary structure of the either pre-miRNA or pri-miRNA *in silico*. Three variants met with the selection criteria: rs376491654 (pri-miR-335), rs755634302 (miR-497), and rs190708267 (pri-miR-155).

**Figure 1:**
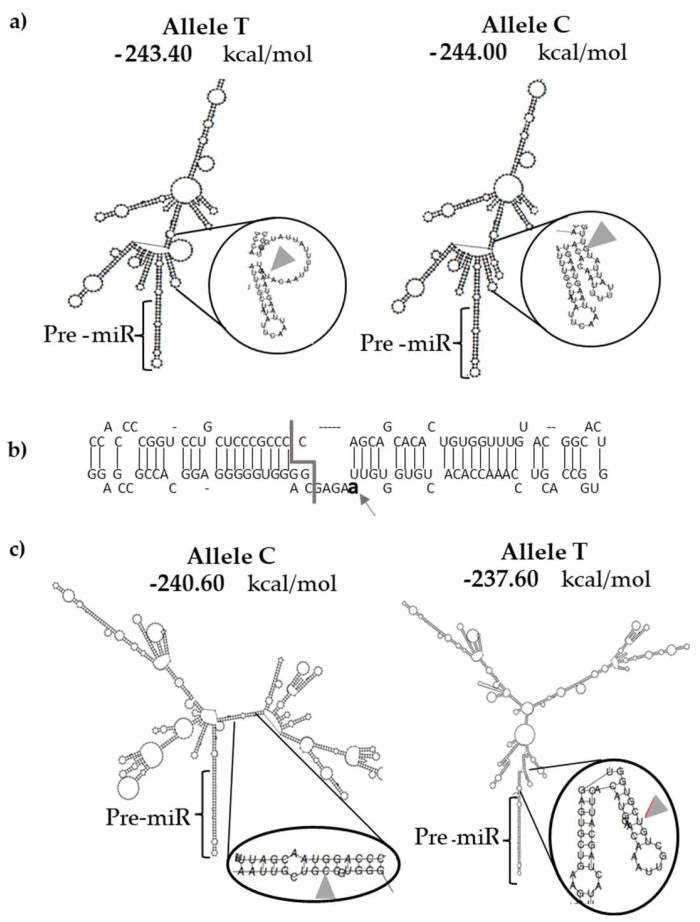
Secondary structure modeling of pri-miRNAs and their genetic variants. (**a**) Structure of pri-miR-335 with the T and C alleles using the RNAfold program. The variant is located at 13 bp of the pre-miRNA. The grey arrowheads indicate the position of rs376491654. (**b**) A schematic of the pre-miR-497 obtained from miRBase is observed, in which the mature 5′ strand and 3′ transient strand are shown. The rs755634302 variant corresponds to an adenine insertion (grey arrow), which is located 3 bp from the Drosha cleavage site (grey line). (**c**) Structure of pri-miR-155 with alleles C and T using the RNAfold program. The rs190708267variant is located 43 bp of the flanking region of the 3′ end of the pre-miRNA-155 and its position is indicated with grey arrowheads.

The variant rs376491654 corresponds to a substitution of T for C, located 13 bp from the 3′ end of the pre-miR-335. After in silico modeling of both alleles, a significant change in the secondary structure was observed. A segment of the flanking ssRNA at the 3′ end was eliminated due to the minor allele frequency (MAF) and produced a new stem (Figure 1a). To genotype this variant, a TaqMan assay was used to analyze 440 BRCA 1/2 negative BC cases and 1031 healthy controls. The homozygous CC genotype was not identified in any of the groups, while the heterozygous (TC) was detected in two of the 440 BC cases and none of the controls. The MAF presented a higher frequency in cases versus controls, however, the difference was not significant (OR = 11.73 [95% CI 0.56–244.95], *p* = 0.089). Thus, the rs376491654-C allele is not associated with the risk of developing BC (Table 2).

The variant rs755634302 corresponds to the insertion of adenine at position 83_84 in the passing strand of the miR-497, located 3 bp of the Drosha cutting site. Upon in silico modeling of the pre-miRNA structure with the insertion, an increased size of an internal loop was observed (Figure 1b). A total of 440 BC samples and 500 controls were genotyped. The homozygous insert genotype was not identified in any of the groups, while the heterozygous was detected in one case, and none of the analyzed controls. The difference between the two groups was not significant (OR = 3.41 [95% CI 0.13–83.9], *p* = 0.468), indicating that the insertion was not associated with the risk for BC (Table 2).

Finally, the variant rs190708267 corresponds to a substitution of C for T located at the 3’ extreme, 43 bp from the pre-miR-155 end. Upon in silico modeling of both alleles, it was observed that the T allele deletes a CG base pairing generating an internal loop, which dramatically changes the pri-miR-155 structure with a consequent change of free energy from −240.60 (C allele) to −237.60 kcal/mol (T allele) as depicted in Figure 1c. Using the TaqMan assay, the rs190708267 was genotyped in 440 BC cases and 1031 healthy controls. Table 2 shows the distributions of genotypes and allele frequencies of the variants in the whole sample set. The genotypic frequency of rs190708267 in the control group was found to be 100% CC. The heterozygous genotype was present only in 0.9% of the BC cases, and the homozygous risk allele (TT) was not detected in cases or controls. The MAF (allele T) had significantly higher frequency in cases versus controls (OR = 21.17 [95% CI 1.138–394.06], *p* = 0.0079). This strongly suggests that rs190708267-T allele is associated with an increased risk for BC.

As shown in Table 2, four cases with CT genotypes were identified among the analyzed samples. These women belong to four families, respectively, with a history of 3 or more cases of BC and with early diagnosis of disease. The average age of diagnosis was 37 years (between 24–50 years). In addition, ovarian, skin, lung, thyroid, and colon cancer were among the other types of cancer diagnosed in these families.

### 2.3. The miR-155-T Allele Increases Levels of the Mature miR-155-5p in BC Cells

**Figure 2:**
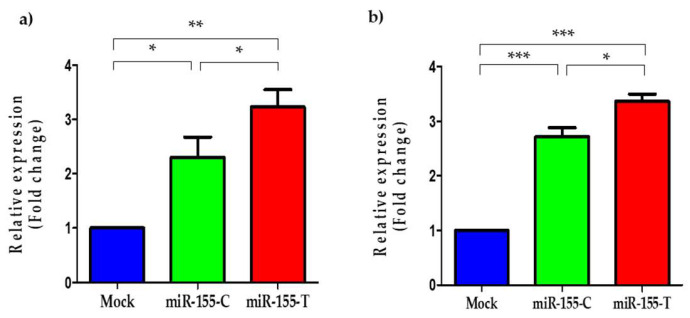
Mature miR-155-5p levels in BC cells transfected with rs190708267. BC cell lines were transfected with miR-155-C, miR-155-T, or mock vectors as indicated and grown for 24 h in complete media. Quantification by TaqMan of the relative levels of the mature form of miR-155-5p in MDA-MB-231 (**a**) and MCF-7 (**b**) cell lines. Data were normalized to RNU6 and expressed in the logarithmic scale. The plotted values indicate the average of three independent experiments ± SD. The asterisks show the significance as indicated. * *p* < 0.05; ** *p* < 0.01; *** *p* < 0.001.

Using different in vitro assays in two BC cell lines (MDA-MB-231 and MCF-7), we assessed the functional effects of the SNVs associated with the risk of developing familial BC at a cellular level. Both cell lines were transiently transfected to overexpress each allele. As rs190708267 localized 43 bp of the pre-miR-155, we hypothesized whether it could alter the processing and, as a consequence, the levels of the mature miR-155-5p. As expected, the T allele significantly increased the levels of mature miR-155-5p in both BC cell lines (Figure 2a,b). Despite the C allele also increasing the mature miR-155-5p levels in both BC cell lines, the presence of the T allele in the flanking region of the pre-miR-155 significantly promoted higher levels of this miRNA, suggesting that the variant rs190708267 could alter its biogenesis and processing.

### 2.4. Proliferation and Resistance to Cisplatin Are Promoted by the miR-155-T Allele

**Figure 3:**
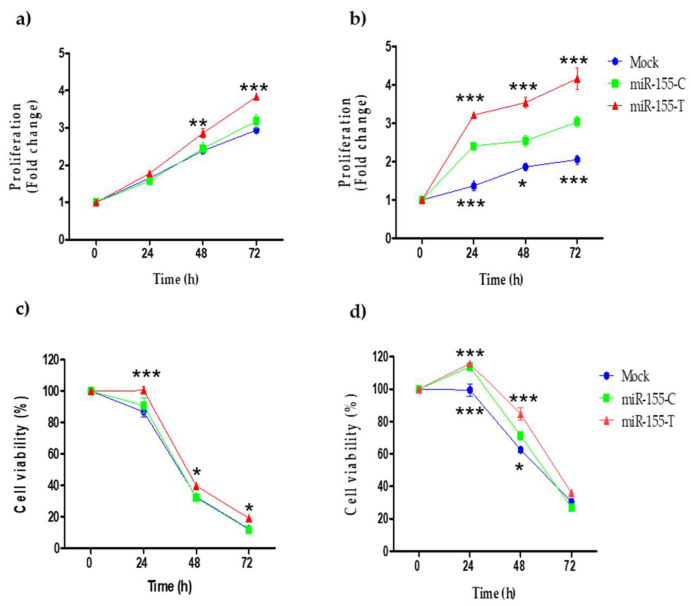
Effect of the miR-155-T allele in proliferation and drug resistance. BC cell lines were transfected with miR-155-C, miR-155-T, or mock vectors, as indicated and grown for 72 h in complete media. Proliferation of MDA-MB-231 (**a**) and MCF-7 (**b**) cells was measured by MTS assay. The graphs show the fold change of absorbance reads at 490 nm at each time. Viability of MDA-MB-231 (**c**) and MCF-7 (**d**) transfected cells upon treatment with 20 μM cisplatin during 72 h. The graphs show the percentages of absorbance reads at 490 nm as compared to time 0. The plotted values indicate the average of three independent experiments ± SD. Significances between the T allele and mock (placed above), as well as between the C allele and mock (placed below), are shown. * *p* < 0.05; ** *p* < 0.01; *** *p* < 0.001.

Several studies have reported that the overexpression of miR-155 in different cell lines promotes cell proliferation [17,24]. Therefore, we evaluated the effect of the T allele on the proliferation of BC cell lines. Figure 3a shows that 48 h after transfection, MDA-MB-231 cells overexpressing miR-155-T proliferated significantly more than cells overexpressing the C allele. In MCF-7 cells, the increase in proliferation in the presence of the T allele was even more dramatic and appeared at 24 h post-transfection (Figure 3b). Previous investigations have associated resistance to various chemotherapeutics with the overexpression of miR-155 in different cancers [18,19,25,26,27,28,29]. Thus, we assessed the effect of the T risk allele on the resistance to cisplatin (20 μM), a drug commonly used in aggressive BCs. As seen in Figure 3c,d, transfection of the rs190708267-T allele conducted more resistance to cisplatin compared to the allele C and mock cells in both MDA-MB-231 and MCF-7 cell lines, respectively.

### 2.5. The miR-155-T Allele Favors Migration and Expression of EMT-Marker Genes

**Figure 4:**
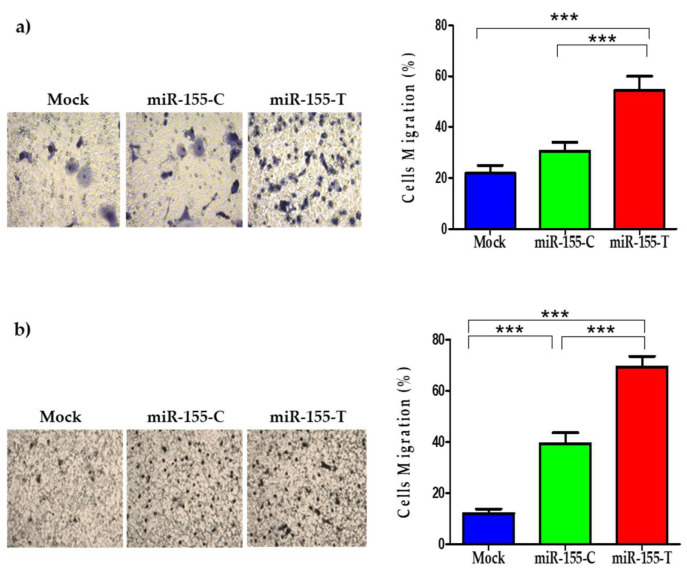
Migration capacity of BC cells expressing the miR-155-T allele. BC cell lines were transfected with miR-155-C, miR-155-T, or mock vectors, as indicated and grown for 24 h in complete media. Migration was evaluated at 8 h of incubation in a transwell assay. Pictures of 10 different fields were taken, and the number of cells was counted for each condition. Representative images of crystal violet-stained cells at 20× magnification (left panels). Relative quantification of migrated cells (right) in which graphs show the average ± the standard error of migrated MDA-MB-231 (**a**) and MCF-7 (**b**) cells. The plotted values indicate the average of three independent experiments ± SD. *** *p* < 0.001.

Migration is a fundamental step in the process of metastasis. Therefore, we studied whether the T allele promotes migration in BC cell lines. The results showed that overexpression of miR-155-T significantly increased the migratory capacity of both cell lines (Figure 4a,b).

**Figure 5:**
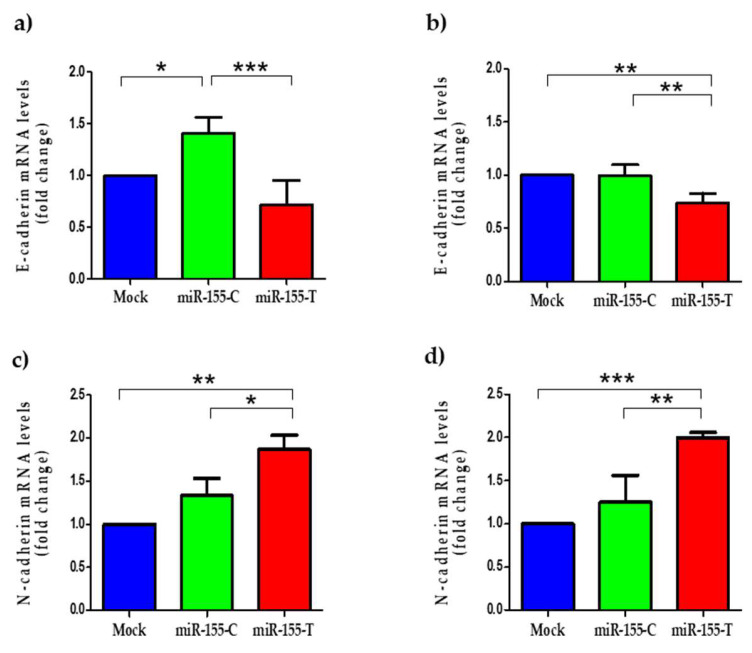
Effect of the miR-155-T allele on mRNA levels of EMT-related genes. BC cell lines were transfected with miR-155-C, miR-155-T, or mock vectors as indicated and grown for 24 h in complete media. The graphs show the relative E-cadherin mRNA levels in MDA-MB-231 (**a**) and MCF-7 (**b**) cell lines. Relative N-cadherin mRNA levels in MDA-MB-231 (**c**) and MCF-7 (**d**) cell lines. The level of GAPDH mRNA was used as a reference. The plotted values indicate the average of three independent experiments ± SD. * *p* < 0.05; ** *p* < 0.01; *** *p* < 0.001.

Besides, to migrate, tumor cells need to change their morphology from an epithelial cell type to a mesenchymal phenotype, a process known as epithelial-to-mesenchymal-transition or EMT [30,31,32]. This process allows cancer cells to migrate through the extracellular matrix and invade the surrounding microenvironment. Thus, we assessed whether the miR-155-T allele affects the expression of EMT markers, such as E-cadherin and N-cadherin. As expected, when mRNA levels of these EMT markers were measured in our cells, we found significant decreased E-cadherin levels (Figure 5a,b), an epithelial marker, increased N-cadherin levels (Figure 5c,d), and a mesenchymal marker in both BC lines expressing the miR-155-T allele.

### 2.6. The miR-155-T Allele Modulates Some Canonical Wnt Pathway Regulators

**Figure 6:**
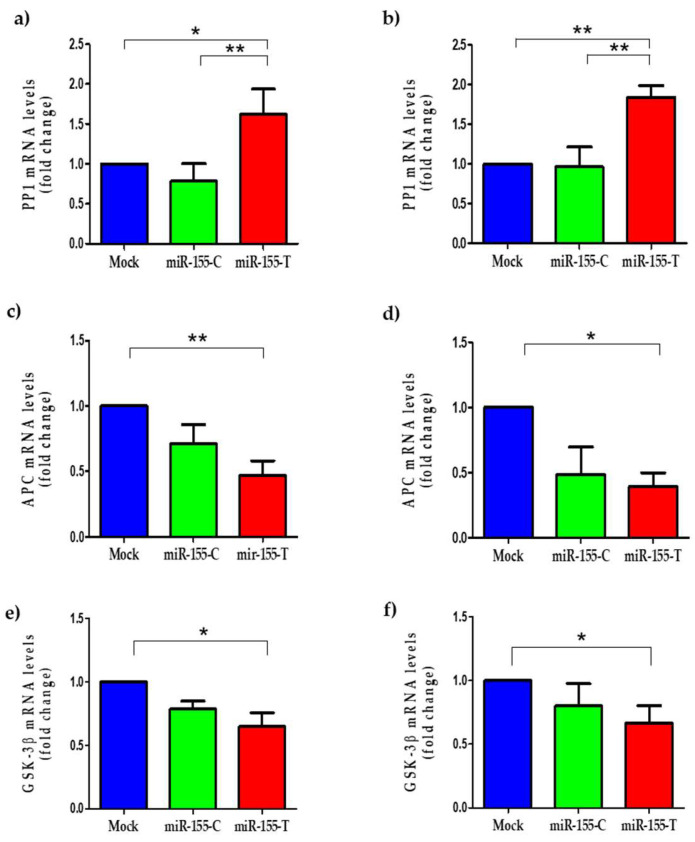
Regulation of canonical Wnt pathway-related elements by the miR-155-T allele. MDA-MB-231 (**a**,**c**,**e**) and MCF-7 (**b**,**d**,**f**) BC cell lines were transfected with miR-155-C, miR-155-T, or mock vectors as indicated and grown for 24 h in complete media. The graphs show the relative mRNA levels of PP1 (**a**,**b**), APC (**c**,**d**), and GSK-3β (**e**,**f**). The levels of GAPDH mRNA were used as a reference. The plotted values indicate the average of three independent experiments ± SD. * *p* < 0.05; ** *p* < 0.01.

More than 500 genes are known as possible targets for the miR-155-5p, so deregulation of its expression could have a significant impact in the expression of a large number of these genes [16,33,34,35,36]. An in silico analysis was performed to predict target genes using the TargetScan Human program available at www.targetscan.org (accessed on 2 June 2020) [37]. Notably, several regulators of the canonical Wnt signaling pathway, including PP1, APC, and GSK-3β, are modulated by the miR-155 [35,36,38] (Figure S1). The canonical Wnt pathway is activated in more than 50% of patients with BC and is associated with worse survival in these patients [39]. To assess the alteration in the expression levels of the mentioned targets, the relative levels of their mRNAs were quantified by RT-qPCR. Transcript levels of PP1, a phosphatase that plays an essential role in the stabilization of β-catenin, were found to be significantly increased in those cells that overexpress the miR-155-T allele (Figure 6a,b). On the other hand, as seen in the cells overexpressing the miR-155-T allele, the mRNA levels of the negative regulators of the canonical Wnt signaling pathway, APC (Figure 6c,d), and GSK-3β (Figure 6e,f) tended to be lower in both BC cell lines. However, once compared only to the mock cells (i.e., empty vector transfected), the mRNA levels were significantly lower in the cells overexpressing the miR-155-T allele.

## 3. Discussion

Many studies have investigated whether variants present in miRNA genes could affect their expression by interfering with their processing and thus contribute substantially to the risk and progression of BC [40]. In the current work, from 11 miRNA genes sequenced, 17 variants were found, of which only one is located in the mature miRNA (the passing strand of miR-497) and 16 variants in the flanking sequences of the pre-miRNA ends (i.e., pri-miRNAs). One of them, rs190708267, corresponding to a substitution of C for T located 43 bp at the 3’ extreme end of pre-miR-155, was associated with an increased risk for BC.

The 1000 Genomes Project Phase 3 database reports two carriers of the rs190708267-T allele in the complete set of samples (n = 2504, 0.1%). When comparing the results of the BC group from the current study (4 carriers out of 440 patients, 0.5%), the *p* value by the Fisher’s exact test was 0.002173, which was a significant difference. All the patients were unrelated (i.e., different families) in our study. Unfortunately, we did not have DNA samples of the other family members of the four BC patients which are carriers of rs190708267-T allele, and thereby we were not able to perform a segregation analysis.

We focused our search on the rs190708267 variant, which was the one proposed as associated with BC in our cohort. We did not find this variant (chr21:25574088-25574088, according to GRCh 38 reference sequence) in the TCGA database (mined using UCSC Xena <xena.ucsc.edu (accessed on 18 November 2022)>) as a somatic mutation, not only in the BC dataset (TCGA-BRCA, n = 1247 samples) but in none of the tumors deposited in the TCGA Pan-Cancer (PANCAN) database (n = 12,839 samples). This mutation was also not found in the Catalogue Of Somatic Mutations In Cancer (COSMIC v96) database.

No variants were found in the sequence of the seed region. A possible explanation to this can be the fact that miRNA genes are very evolutionarily conserved among species [41]. The above is consistent with several studies reporting that the density of SNPs is significantly higher in the flanking regions of the pri-miRNAs than in the pre-miRNAs and is even lower in the seed region of mature miRNAs. Several studies have shown that variants present in the sequence of miRNA genes can alter their biogenesis, affecting the expression levels of mature miRNA directly or affecting its functionality and interaction with its target mRNAs, consequently contributing to susceptibility to diseases such as cancer [42]. The miR-155 gene is considered an oncogene since its overexpression has been associated with a wide variety of cancers, such as lung, gastric, pancreatic, thyroid, B-cell lymphoma, and BC [5,15,29,43,44]. Overexpression of miR-155 has been associated with metastatic events and invasive properties in BC tumors [17,45,46,47,48]. Chen et al., 2012 [49], reported that the increase in miR-155 in BC is associated with high-grade tumors, more advanced stages, and lymph node metastases. Additionally, the levels of this miRNA correlate negatively with disease-free survival, suggesting the carcinogenic potential of miR-155 [49].

Our in silico analysis of rs190708267 variant revealed that the substitution eliminates one G:C base pairing, generating a protrusion in the pri-miRNA, altering the minimum free energy, and thus modifying the stability of the secondary structure of the pri-miR-155. In the current work, we found that when the T allele is present (i.e., miR-155-T), mature miR-155 levels are significantly higher compared to those cells that overexpress the C allele. These results are in accordance with other studies, showing that mutations present in the pre-miRNA or pri-miRNA can influence stability in miRNA processing, thus explaining the differences seen between the C and T alleles [50,51]. This leads us to suggest that rs190708267 would affect Drosha’s processing of the pri-miR-155.

Several studies have reported that miR-155 overexpression increased proliferation in colon, lung, cervical, and BC cells [16,33,52,53,54]. These data are in line with our results where increased levels of miR-155-T, due to the presence of the risk allele, promoted an increase in proliferation in BC cells. Likewise, we found that miR-155-T allele is also associated with resistance to cisplatin in BC cells. It has been reported that miR-155 increases resistance to different drug-based therapies. For example, the aberrant expression of this miRNA induces chemoresistance to tamoxifen, cisplatin, paclitaxel, and doxorubicin in lung cancer, leukemia, endocervical adenocarcinoma, and BC [18,19,25,27]. In the work of Van Roosbroeck et al., 2017 [18], the effect of miR-155 on chemoresistance in vivo in an orthotopic model of lung cancer was evaluated. They found that high levels of this miRNA promoted resistance to cisplatin in tumors, while treatment with the anti-miR-155 reduced the growth and size of tumors. Additionally, they observed that the combined treatment of anti-miR-155 and cisplatin completely reversed chemoresistance [18].

Several studies have shown that high levels of miR-155 in BC promote the migratory capacity of cancer cells, which is also consistent with our results. The miR-155-T allele significantly augmented the migration, decreased levels of E-cadherin mRNA, and increased N-cadherin mRNA in both BC cell lines. A similar observation was reported in colon cancer cells, where a decrease in E-cadherin was observed, concomitant with increased migration and invasion in cells that overexpressed the pre-miR-155, suggesting that this miRNA is involved in the expression of proteins that participate in EMT [53]. Although we cannot state at this time that these mRNAs are indeed direct targets of miR-155-T, our findings were significant enough to suggest that an EMT process occurs in both BC cells.

The miRNAs have numerous targets, therefore, variations in the expression of a miRNA can have significant consequences for the expression of various oncogenes and tumor suppressors involved in aggressiveness [47]. Up to date, no association has been reported between miR-155 and canonical Wnt pathway in BC. However, overexpression of miR-155 in thyroid cancer cell lines decreased APC expression at a transcript and protein level, increasing proliferation and tumorigenic capacity, suggesting miR-155 as a regulator of this pathway [52]. Here, we found that the mRNA levels of the negative regulators of the Wnt pathway, APC and GSK-3β, were lower in both BC cell lines, while those of the PP1 increased significantly upon expression of the miR-155-T allele. Further studies would be necessary to determine if once β-catenin was found free in the cytoplasm of these cells and then translocate it to the nucleus and activate its target genes, which participate in proliferation, migration, and drug resistance, explaining our observed findings.

To date, there are no standards and guidelines to interpret the pathogenicity of a variant present in miRNA genes such as those developed by the American College of Medical Genetics (ACMG) for variants present in protein-coding genes [55]. However, using this guide as a benchmark, we assessed the probability that rs190708267 is a putative pathogenic germline variant that could contribute to the risk of developing early-onset BC. The criteria employed were allele frequency, degree of evolutionary conservation, in silico structure prediction, and functional effects *in vitro*.

The rs190708267-T has a frequency of 0.0045 in Chilean BC patients (n = 440), while this variant was not detected in the control population of healthy women. The degree of evolutionary conservation of the ancestral allele was analyzed. We did an alignment of the nucleotide sequence of pre-miR-155 and flanking region containing the rs190708267 of different species (Figure S2). Interestingly, it was found that within the poorly conserved region, the ancestral C allele is highly conserved. Besides, according to our results, women carrying the rs190708267-T allele had an early-onset diagnosis with an average age of 37 years (between 24 and 50 years). In one of the carrier families, four women carry the rs190708267-T allele. It was observed that the variant co-segregates with BC (Figure S3). One of them (with breast and ovarian cancer) died due to metastasis. Together, these facts led us to propose that rs190708267-T is likely a pathogenic variant.

## 4. Materials and Methods

### 4.1. BC Population Study

Patients with hereditary BC were recruited from public and private health services located in Santiago of Chile. All were diagnosed with breast adenocarcinoma according to the histopathological report. All participants gave their written informed consent and completed a questionnaire related to their medical and reproductive history, ethnicity, and risk factors. Their genealogy was reconstructed. This research was approved by the Institutional Review Board, Facultad de Medicina, Universidad de Chile (Fondecyt 1150117). All methods were performed in accordance with the relevant guidelines and regulations. The selection criteria for the patient’s inclusion and analysis of mutations present in the BRCA 1 and 2 genes were previously reported in De Mayo et al. [56]. In total, 440 cases negative for BRCA 1/2 gene mutations were selected for this study.

### 4.2. Selection and Sequencing of Deregulated microRNA Genes in BRCA Negative Patients

We searched the literature to select miRNAs that met the following criteria: (i) are commonly deregulated in BC; (ii) target mRNAs associated with BC; (iii) are involved in drug resistance, and (iv) have an unknown mechanism of aberrant expression. The selected miRNAs were miR-10b, miR-21, miR-125a, miR-145, miR-155, miR-497, miR-195, miR-221, miR-222, miR-335, and miR-520c. Their genes were sequenced by Sanger in DNA of 100 patients (from the 440 BRCA 1/2 negative) whose families had a strong family history (Table S1). Primers were designed using the PRIMER3 software (http://bioinfo.ut.ee/primer3-0.4.0/primer3/ accessed on 1 March 2016), which included the sequence of the pre-miRNA ± 200 bp flanking sequence at both ends. The sequences and Tm of the primers, as well as the sizes of the amplified sequences, are in Table S3.

The PCR was carried out with 40 ng DNA, 1X Buffer, 2 mM dNTP, 50 mM MgCl_2_, 10 pmol primers, and 1.5 U DNA polymerase (Biotools, Madrid, Spain). The protocol was 94 °C for 5 min, followed by 30 cycles at 94 °C for 30 s, 60 °C for 30 s, 72 °C for 30 s, and finally an extension of 7 min at 72 °C. Amplification was verified on a 2% agarose gel using 5 µL SYBR Safe DNA Gel Stain (Invitrogen, Carlsbad, CA, USA) for visualization of the DNA bands. The PCR products were Sanger-sequenced (Macrogen Inc., Seoul, Korea). Obtained electrophoretograms were analyzed and assembled using Chromas Pro 1.5 (Technelysium Pty Ltd., South Brisbane, Australia) to identify the variations present in the studied genes.

### 4.3. In Silico Analysis of Secondary Structures of the Pri-microRNAs

To assess how the identified variants could alter the secondary structure of each miRNA, the pre-miRNA plus 500 bp of the flanking sequences (±1100 bp) were modeled with the common or with the less frequent variant to compare whether the latter affects the secondary structure of pre-miRNA. We used the RNAfold program available at http://rna.tbi.univie.ac.at/cgi-bin/RNAfold.cgi (accessed on 15 October 2020).

### 4.4. Genotyping of the Selected Variants

For the association study, 440 cases of familial BC without *BRCA1*/*BRCA2* genes mutations and 1031 controls were genotyped, using TaqMan^®^ SNP Genotyping Assays (Applied Biosystems, Foster City, CA, USA) to genotype rs190708267 (miR-155) and rs376491654 (miR-335) variants. The reaction was performed with 5 ng of genomic DNA and 1X of TaqMan^®^ Genotyping Master Mix (ThermoFisher, Waltham, MA, USA) according to the manufacturer’s instructions. The reaction was carried out in a StepOnePlus real-time PCR system (Applied Biosystems, Foster City, CA, USA), starting with 10 min at 95 °C, followed by 40 cycles each at 92 °C for 15 s and 60 °C for 1 min. DNA samples with confirmed genotype by Sanger sequencing were used as controls. Alleles were assigned using StepOne V2.2 software (Applied Biosystems, Foster City, CA, USA).

The rs755634302 (miR-497) was genotyped in 440 cases with familial BC and 500 controls using the Restriction Fragment Length Polymorphism (RFLP) technique. The PCR product was obtained using the same protocol as described above for sequencing and digested with the restriction enzyme *MseI* (New England Biolabs, Ipswich, MA, USA) according to the manufacturer’s instructions. The insert allele results in fragments of 426 bp and 171 bp, while the other allele is not digested (597 bp). The digestion products were resolved by agarose (1.5%) electrophoresis.

### 4.5. Cloning of the Polymorphic miR-155 Sequences

Two 500 bp fragments (gBlocks IDT) containing the pre-miRNA-155 sequence were synthesized, one with the C allele and the other with the T allele. A flanking sequence of approximately 100 bp on either side of the pre-miRNA has been shown to be sufficient for the biogenesis of miRNAs [57]. Therefore, for each fragment, approximately 200 bp were included at each end. Both fragments were inserted into a pcDNA 3.3 expression vector using pcDNATM 3.3-TOPO^®^TA Cloning Kit (ThermoFisher, Waltham, MA, USA) following the manufacturer’s instructions. Insertion and orientation of the fragment were confirmed by Sanger sequencing (Macrogen Inc., Seoul, Korea), generating the vectors miR-155-C, miR-155-T, and the mock control (empty vector).

### 4.6. Cell Cultures and Transfection of Polymorphic microRNAs

Two BC cell lines were used for in vitro analyses: the triple-negative molecular subtype (HER2^−^, ER^−^, and PR^−^) MDA-MB-231 cell line and the sporadic BC cell line MCF-7 (invasive ductal carcinoma, HER2^−^, ER^+^, and PR^+^). Both cell lines were grown at 37 °C and 5% CO_2_ in DMEM medium supplemented with 10% fetal bovine serum, including 100 units/mL of penicillin G and 10 µg/mL of streptomycin.

Transfection of both cell lines was performed in order to express the miR-155-C or miR-155-T variants. On the first day, 6 × 10^5^ cells were seeded in 6 cm dishes and grown for 24 h. On the second day, a tube with the transfection reaction was prepared using 12 µL of Lipofectamine^®^ 2000 (ThermoFisher, Waltham, MA, USA) and 38 µL of Opti-MEM^®^ serum reduced medium (ThermoFisher, Waltham, MA, USA) for each plate. In parallel, 2 µg of the purified vector was mixed with Opti-MEM to reach the final volume of 50 µL. Subsequently, both preparations were mixed, incubated at room temperature (25 °C) for 30 min, and added to washed cells. The transfection efficiency (between 60–70%) was determined using the pEGFP vector (at the same concentration), which only expresses the green fluorescent protein (GFP).

### 4.7. RNA Extraction and RT-qPCR

Total RNA from transfected cells was extracted using the ENZA Total RNA Kit I (Omega Biotek, GA, USA) following the manufacturer’s instructions. The RNA was then treated with DNAse using the DNase kit (Ambion, Life Technologies, Carlsbad, CA, USA) and quantified using the NanoQuant Infinite M200 pro spectrophotometer (Tecan, Hombrechtikon, Switzerland). Subsequently, using 600 ng of RNA, the cDNA was synthesized with the cDNA AFFINITY Script qPCR kit (Agilent Technologies, TX, USA) using the following protocol: 5 min at 25 °C, 45 min at 42 °C, and 5 min at 95 °C. The quantitative PCR was performed in a StepOnePlus Real-Time PCR System using SYBR Green PCR Master Mix kit (ThermoFisher, Waltham, MA, USA) according to the manufacturer’s instructions. Briefly, we did 95 °C for 30 s, then 40 cycles 95 °C for 5 s and 60 °C for 30 s, ending with a melting curve of 95 °C for 15 s, 60 °C for 1 min, and 95 °C for 15 s. *GAPDH* gene was used as a normalizing gene. All primers are listed in Table S3. To measure the relative expression levels of the mature miR-155, the cDNA of the miR-155 and the RNU6 reference gene was synthesized using 100 μg of total RNA, TaqMan^®^ MicroRNA Assay specific primers (miR-155-5p ID 000479, RNU6 ID 0001973) and TaqMan^®^ MicroRNA Reverse Transcription Kit (Applied Biosystems, Foster City, CA, USA) following manufacturer’s instructions. For the qPCR, 2 μL of cDNA, TaqMan^®^ Universal Master Mix II master mix, without UNG (ThermoFisher, Waltham, MA, USA), the TaqMan probe (20X) included in the TaqMan^®^ MicroRNA Assay was used. The PCR reaction was carried out according to the following protocol: 95 °C for 10 min, then 40 cycles at 95 °C for 15 s and 60 °C for 1 min, ending with an extension at 60 °C. Quantifications were performed in triplicates and the relative concentrations were expressed using the 2^−ΔΔCT^ method [58].

### 4.8. Transwell Migration Assay

For the 3D migration assays, the outer base of the transwell upper chamber (Corning, Mexico) was incubated with 2 µg/mL fibronectin for 16 h at 4 °C. Subsequently, 4 × 10^4^ of transfected cells were seeded in DMEM medium without serum in the transwells, and 500 µL of DMEM medium with serum was added in the wells. Cells were incubated at 37 °C for 8 h. The migrating cells were fixed and stained with 0.05% crystal violet and 20% methanol dissolved in 0.15 M NaCl for 1 h at room temperature. Non-migrating cells were removed with a cotton swab. For quantification, pictures of 10 different fields were taken with Nikon Eclipse TS100 Inverted Microscope (20× magnification), and the number of cells was counted for each condition.

### 4.9. Cell Proliferation and Cisplatin Resistance

The number of viable proliferating cells was determined using the CellTiter 96^®^ AQueous Non-Radioactive Cell Proliferation Assay MTS kit (Promega, Madison, WI, USA). Twenty-four h post-transfection, 1 × 10^4^ cells were seeded in 96-well plate in quadruplicates, and cell proliferation was measured every 24 h. To measure drug resistance, 20 μM cisplatin (vehicle DMSO as control) was added in the plate at the start point. Then, the MTS reagent was added according to the manufacturer’s instructions, and cells were incubated for 3 h at 37 °C. Absorbance was read at a wavelength of 490 nm on a NanoQuant Infinite M200 pro spectrophotometer (Tecan, Hombrechtikon, Switzerland).

### 4.10. Statistical Analysis

The results were plotted as the mean ± SEM from at least three independent experiments. Statistical analyses were performed with GraphPad Prism version 6.0 program (GraphPad, San Diego, CA, USA) by using ANOVA as first test and further a Tukey’s Multiple Comparison as post-test. In the case-control study, a Fisher’s exact was used to test the association of SNV with BC. The Odds ratio (OR) and 95% confidence interval (CI) were calculated to estimate the strength of association of SNV with BC. Analyses were done using Stata 8.2 for Windows intercooler (StataCorp, TX, USA). A *p* value of less than 0.05 was considered significant.

## 5. Conclusions

We found that the presence of rs190708267-T increases the levels of mature miR-155. Our in silico analysis points out that the allele T can affect the secondary structure of the pri-miRNA, suggesting an alteration of its processing. This variant also promoted cellular in vitro traits related to aggressiveness in BC, such as proliferation, migration, and chemoresistance to cisplatin. Altogether, taking into consideration our allele frequency, degree of evolutionary conservation, in silico functional prediction, and functional cell assays, we propose to the rs190708267-T at pri-miR-155 as a pathogenic germline variant that contributes to the aggressiveness of BRCA 1/2 negative BC.

## Figures and Tables

**Table 1:** Germline variants found in sequenced miRNA genes.

Gene	Chromosomal Location	rsID	Nucleotide Change	Relative Position
hsa-miR-10b	Chr.2	rs1867863	NC_000002.12:g.176150242G>T	61 nt 5′upstream from pre-miR-10b (ENST00000385011.1)
		rs138423463	NC_000002.12:g.176150514A>G	102 nt 3′downstream from pre-miR-10b (ENST00000385011.1)
		ND	ND	189 nt 3′downstream from pre-miR-10b (ENST00000385011.1)
		ND	ND	199 nt 3′downstream from pre-miR-10b (ENST00000385011.1)
hsa-miR-21	Chr.17	rs570199250	NC_000017.11:g.59841173_59841174insT	99 nt 5′upstream from pre-miR-21 (ENST00000362134.1)
hsa-miR-125a	Chr.19	rs12976445	NC_000019.10:g.51693200T>C	54 nt 5′upstream from pre-miR-125a (ENST00000385273.1)
		rs372615282	NC_000019.10:g.51693210T>C	44 nt 5′upstream from pre-miR-125a (ENST00000385273.1)
		rs78758318	NC_000019.10:g.51693469G>A	129 nt 3′downstream from pre-miR-125a (ENST00000385273.1)
		rs11881781	NC_000019.10:g.51693559G>C	218 nt 3′downstream from pre-miR-125a (ENST00000385273.1)
hsa-miR-145	Chr.5	*	*	*
hsa-miR-155	Chr.21	rs190708267	NC_000021.9:g.25574088C>T	43 nt 3′downstream from pre-miR-155 (ENST00000385060.1)
hsa-miR-195	Chr.17	rs41283391	NC_000017.11:g.7017569C>T	45 nt 3′downstream from pre-miR-195 (ENST00000385194.1)
hsa-miR-221	Chr. X	rs191213444	NC_000023.11:g.45746349G>T	83 nt 5′upstream from pre-miR-221 (ENST00000385135.1)
hsa-miR-222	Chr. X	*	*	*
hsa-miR-335	Chr.7	rs201306521	NC_000007.14:g.130495936_130495937insT	174 nt 3′downstream from pre-miR-335 (ENST00000362173.1)
		rs3807348	NC_000007.14:g.130496266G>A	62 nt 3′downstream from pre-miR-335 (ENST00000362173.1)
		rs376491654	NC_000007.14:g.130496217T>C	13 nt 3′downstream from pre-miR-335 (ENST00000362173.1)
		rs41272366	NC_000007.14:g.130496224T>A	19 nt 3′downstream from pre-miR-335 (ENST00000362173.1)
hsa-miR-520c	Chr.19	*	*	*
hsa-miR-497	Chr.17	rs755634302	NC_000017.11:g.7017940dup	Nucleotide 83 of mature miR-497 ENST00000385056.1

ND: not described; *: no variants found; Chr: chromosome.

**Table 2:** Genotype and allele frequencies of germ variants in BRCA 1/2 negative BC cases and controls.

SNP ID	Genotype or Allele	Controls (%) *n* = 1031	BC Cases (%) *n* = 440	OR [95% CI]	*p* Value ^a^
rs376491654 (miR-335)	TT	1031 (100)	438 (99.5)	1.0 (ref)	
	TC	0	2 (0.5)	-	
	CC	0	0	-	
	Allele T	2062 (100)	878 (99.7)	1.0 (ref)	
	Allele C	0	2 (0.3)	11.73 [0.56–244.95]	0.0890
rs755634302 (miR-497)	-/-	500 (100)	439 (99.7)	1.0 (ref)	
	InsA/-	0	1 (0.3)	-	
	InsA/InsA	0	0	-	
	Allele -	1000 (100)	879 (99.8)	1.0 (ref)	
	Allele InsA	0	1 (0.2)	3.41 [0.13–83.9]	0.4680
rs190708267 (miR-155)	CC	1031 (100)	436 (99.1)	1.0 (ref)	
	CT	0	4 (0.9)	-	
	TT	0	0	-	
	Allele C	2062 (100)	876 (99.5)	1.0 (ref)	
	Allele T	0	4 (0.5)	21.17 [1.138–394.06]	**0.0079**

BC: breast cancer; OR: odds ratio; CI: confidence interval; -: without insertion; ref: reference; ^a^ Fisher’s exact test.

## Data Availability

All methods in this study can be found in a previous published article of our authority [59].

## Supplementary Materials

The supporting information can be downloaded at: https://www.mdpi.com/article/10.3390/ijms232315418/s1.

## References

[1] Ferlay J., Colombet M., Soerjomataram I., Mathers C., Parkin D.M., Piñeros M., Znaor A., Bray F. (2019). Estimating the global cancer incidence and mortality in 2018: GLOBOCAN sources and methods. Int. J. Cancer.

[2] Ferlay J., Soerjomataram I., Dikshit R., Eser S., Mathers C., Rebelo M., Parkin D.M., Forman D., Bray F. (2015). Cancer incidence and mortality worldwide: Sources, methods and major patterns in GLOBOCAN 2012. Int. J. Cancer.

[3] Couch F.J., Nathanson K.L., Offit K. (2014). Two Decades After BRCA: Setting Paradigms in Personalized Cancer Care and Prevention. Science.

[4] Pharoah P.D.P., Dunning A.M., Ponder B.A.J., Easton D.F. (2004). Association studies for finding cancer-susceptibility genetic variants. Nat. Rev. Cancer.

[5] Iorio M.V., Ferracin M., Liu C.-G., Veronese A., Spizzo R., Sabbioni S., Magri E., Pedriali M., Fabbri M., Campiglio M. (2005). MicroRNA Gene Expression Deregulation in Human Breast Cancer. Cancer Res..

[6] Tahiri A., Aure M.R., Kristensen V.N. (2018). MicroRNA Networks in Breast Cancer Cells. Methods Mol. Biol..

[7] Tekiner T.A., Basaga H. (2013). Role of microRNA deregulation in breast cancer cell chemoresistance and stemness. Curr. Med. Chem..

[8] Sabit H., Cevik E., Tombuloglu H., Abdel-Ghany S., Tombuloglu G., Esteller M. (2021). Triple negative breast cancer in the era of miRNA. Crit. Rev. Oncol./Hematol..

[9] Balkrishna A., Mittal R., Arya V. (2021). Potential Role of miRNA in Metastatic Cascade of Triple-Negative Breast Cancer. Curr. Cancer Drug Targets.

[10] Aigner A. (2011). MicroRNAs (miRNAs) in cancer invasion and metastasis: Therapeutic approaches based on metastasis-related miRNAs. J. Mol. Med..

[11] van Schooneveld E., Wildiers H., Vergote I., Vermeulen P.B., Dirix L.Y., Van Laere S.J. (2015). Dysregulation of microRNAs in breast cancer and their potential role as prognostic and predictive biomarkers in patient management. Breast Cancer Res..

[12] Bertoli G., Cava C., Castiglioni I. (2015). MicroRNAs: New Biomarkers for Diagnosis, Prognosis, Therapy Prediction and Therapeutic Tools for Breast Cancer. Theranostics.

[13] Mashima R. (2015). Physiological roles of miR-155. Immunology.

[14] Hsin J.-P., Lu Y., Loeb G.B., Leslie C.S., Rudensky A.Y. (2018). The effect of cellular context on miR-155-mediated gene regulation in four major immune cell types. Nat. Immunol..

[15] Metzler M., Wilda M., Busch K., Viehmann S., Borkhardt A. (2004). High expression of precursor microRNA-155/BIC RNA in children with Burkitt lymphoma. Genes Chromosomes Cancer.

[16] Zhang C.M., Zhao J., Deng H.Y. (2013). MiR-155 promotes proliferation of human breast cancer MCF-7 cells through targeting tumor protein 53-induced nuclear protein 1. J. Biomed. Sci..

[17] Zuo J., Yu Y., Zhu M., Jing W., Yu M., Chai H., Liang C., Tu J. (2018). Inhibition of miR-155, a therapeutic target for breast cancer, prevented in cancer stem cell formation. Cancer Biomarkers.

[18] Van Roosbroeck K., Fanini F., Setoyama T., Ivan C., Rodriguez-Aguayo C., Fuentes-Mattei E., Xiao L., Vannini I., Redis R.S., D’Abundo L. (2017). Combining Anti-Mir-155 with Chemotherapy for the Treatment of Lung Cancers. Clin. Cancer Res..

[19] Lei C., Wang Y., Huang Y., Yu H., Huang Y., Wu L., Huang L. (2012). Up-regulated miR155 reverses the epithelial-mesenchymal transition induced by EGF and increases chemo-sensitivity to cisplatin in human Caski cervical cancer cells. PLoS ONE.

[20] Han M., Zheng Y. (2013). Comprehensive analysis of single nucleotide polymorphisms in human microRNAs. PLoS ONE.

[21] Sun G., Yan J., Noltner K., Feng J., Li H., Sarkis D.A., Sommer S.S., Rossi J.J. (2009). SNPs in human miRNA genes affect biogenesis and function. RNA.

[22] Ryan B.M., Robles A.I., Harris C.C. (2010). Genetic variation in microRNA networks: The implications for cancer research. Nat. Rev. Cancer.

[23] Bahreini F., Rayzan E., Rezaei N. (2021). microRNA-related single-nucleotide polymorphisms and breast cancer. J. Cell. Physiol..

[24] Kong W., He L., Coppola M., Guo J., Esposito N.N., Coppola D., Cheng J.Q. (2010). MicroRNA-155 regulates cell survival, growth, and chemosensitivity by targeting FOXO3a in breast cancer. J. Biol. Chem..

[25] Bayraktar R., Van Roosbroeck K. (2018). miR-155 in cancer drug resistance and as target for miRNA-based therapeutics. Cancer Metastasis Rev..

[26] Zang Y.-S., Zhong Y.-F., Fang Z., Li B., An J. (2012). MiR-155 inhibits the sensitivity of lung cancer cells to cisplatin via negative regulation of Apaf-1 expression. Cancer Gene Ther..

[27] Gasparini P., Lovat F., Fassan M., Casadei L., Cascione L., Jacob N.K., Carasi S., Palmieri D., Costinean S., Shapiro C.L. (2014). Protective role of miR-155 in breast cancer through RAD51 targeting impairs homologous recombination after irradiation. Proc. Natl. Acad. Sci. USA.

[28] Shen R., Wang Y., Wang C.-X., Yin M., Liu H.-L., Chen J.-P., Han J.-Q., Wang W.-B. (2015). MiRNA-155 mediates TAM resistance by modulating SOCS6-STAT3 signalling pathway in breast cancer. Am. J. Transl. Res..

[29] Yu D.-D., Lv M.-M., Chen W.-X., Zhong S., Zhang X.-H., Chen L., Ma T.-F., Tang J.-H., Zhao J.-H. (2015). Role of miR-155 in drug resistance of breast cancer. Tumour Biol..

[30] Ye X., Weinberg R.A. (2015). Epithelial-Mesenchymal Plasticity: A Central Regulator of Cancer Progression. Trends Cell Biol..

[31] Zhang Y., Weinberg R.A. (2018). Epithelial-to-mesenchymal transition in cancer: Complexity and opportunities. Front. Med..

[32] Peng F., Xiong L., Tang H., Peng C., Chen J. (2016). Regulation of epithelial-mesenchymal transition through microRNAs: Clinical and biological significance of microRNAs in breast cancer. Tumour Biol..

[33] Zhang W., Chen C.J., Guo G.L. (2018). MiR-155 promotes the proliferation and migration of breast cancer cells via targeting SOCS1 and MMP16. Eur. Rev. Med. Pharmacol. Sci..

[34] Jiang S., Zhang H.-W., Lu M.-H., He X.-H., Li Y., Gu H., Liu M.-F., Wang E.-D. (2010). MicroRNA-155 functions as an OncomiR in breast cancer by targeting the suppressor of cytokine signaling 1 gene. Cancer Res..

[35] Zhang Y., Wei W., Cheng N., Wang K., Li B., Jiang X., Sun S. (2012). Hepatitis C virus-induced up-regulation of microRNA-155 promotes hepatocarcinogenesis by activating Wnt signaling. Hepatology.

[36] Prossomariti A., Piazzi G., D’Angelo L., Miccoli S., Turchetti D., Alquati C., Montagna C., Bazzoli F., Ricciardiello L. (2018). miR-155 Is Downregulated in Familial Adenomatous Polyposis and Modulates WNT Signaling by Targeting AXIN1 and TCF4. Mol. Cancer Res..

[37] Agarwal V., Bell G.W., Nam J.-W., Bartel D.P. (2015). Predicting effective microRNA target sites in mammalian mRNAs. eLife.

[38] Zhao J., Feng Y., Yan H., Chen Y., Wang J., Chua B., Stuart C., Yin D. (2014). β-arrestin2/miR-155/GSK3β regulates transition of 5′-azacytizine-induced Sca-1-positive cells to cardiomyocytes. J. Cell. Mol. Med..

[39] Lin S.-Y., Xia W., Wang J.C., Kwong K.Y., Spohn B., Wen Y., Pestell R.G., Hung M.-C. (2000). β-Catenin, a novel prognostic marker for breast cancer: Its roles in cyclin D1 expression and cancer progression. Proc. Natl. Acad. Sci. USA.

[40] Chen J., Jiang Y., Zhou J., Liu S., Gu Y., Jin G., Hu Z., Ma H., Shen H., Dai J. (2017). Genetic Variants in the Promoter Region of. BioMed Res. Int..

[41] Chen K., Rajewsky N. (2006). Deep conservation of microRNA-target relationships and 3’UTR motifs in vertebrates, flies, and nematodes. Cold Spring Harb. Symp. Quant. Biol..

[42] Fang C., Zeng H., Li A., Xu X., Long X. (2015). Association of the pri-miR-124-1 rs531564 polymorphism with cancer risk: A meta-analysis. Mol. Clin. Oncol..

[43] Yu H., Xu W., Gong F., Chi B., Chen J., Zhou L. (2017). MicroRNA-155 regulates the proliferation, cell cycle, apoptosis and migration of colon cancer cells and targets CBL. Exp. Ther. Med..

[44] Shao C., Yang F., Qin Z., Jing X., Shu Y., Shen H. (2019). The value of miR-155 as a biomarker for the diagnosis and prognosis of lung cancer: A systematic review with meta-analysis. BMC Cancer.

[45] Iorio M.V., Croce C.M. (2012). Causes and consequences of microRNA dysregulation. Cancer J..

[46] Volinia S., Calin G.A., Liu C.G., Ambs S., Cimmino A., Petrocca F., Visone R., Iorio M., Roldo C., Ferracin M. (2006). A microRNA expression signature of human solid tumors defines cancer gene targets. Proc. Natl. Acad. Sci. USA.

[47] Zeng H., Fang C., Nam S., Cai Q., Long X. (2014). The clinicopathological significance of microRNA-155 in breast cancer: A meta-analysis. BioMed Res. Int..

[48] Brown C.Y., Dayan S., Wong S.W., Kaczmarek A., Hope C.M., Pederson S.M., Arnet V., Goodall G.J., Russell D., Sadlon T.J. (2018). FOXP3 and miR-155 cooperate to control the invasive potential of human breast cancer cells by down regulating ZEB2 independently of ZEB1. Oncotarget.

[49] Chen J., Wang B.C., Tang J.H. (2012). Clinical significance of microRNA-155 expression in human breast cancer. J. Surg. Oncol..

[50] Suresh P.S., Venkatesh T., Tsutsumi R. (2016). In silico analysis of polymorphisms in microRNAs that target genes affecting aerobic glycolysis. Ann. Transl. Med..

[51] Kunej T., Godnic I., Horvat S., Zorc M., Calin G.A. (2012). Cross talk between microRNA and coding cancer genes. Cancer J..

[52] Zhang X., Li M., Zuo K., Li D., Ye M., Ding L., Cai H., Fu D., Fan Y., Lv Z. (2013). Upregulated miR-155 in papillary thyroid carcinoma promotes tumor growth by targeting APC and activating Wnt/β-catenin signaling. J. Clin. Endocrinol. Metab..

[53] Zhang G.-J., Xiao H.-X., Tian H.-P., Liu Z.-L., Xia S.-S., Zhou T. (2013). Upregulation of microRNA-155 promotes the migration and invasion of colorectal cancer cells through the regulation of claudin-1 expression. Int. J. Mol. Med..

[54] Lao G., Liu P., Wu Q., Zhang W., Liu Y., Yang L., Ma C. (2014). Mir-155 promotes cervical cancer cell proliferation through suppression of its target gene LKB1. Tumour Biol..

[55] Richards S., Aziz N., Bale S., Bick D., Das S., Gastier-Foster J., Grody W.W., Hegde M., Lyon E., Spector E. (2015). Standards and guidelines for the interpretation of sequence variants: A joint consensus recommendation of the American College of Medical Genetics and Genomics and the Association for Molecular Pathology. Genet. Med..

[56] De Mayo T., Ziegler A., Morales S., Jara L. (2018). Identification of a Rare Germline Heterozygous Deletion Involving the Polycistronic miR-17–92 Cluster in Two First-Degree Relatives from a BRCA 1/2 Negative Chilean Family with Familial Breast Cancer: Possible Functional Implications. Int. J. Mol. Sci..

[57] Duan R., Pak C., Jin P. (2007). Single nucleotide polymorphism associated with mature miR-125a alters the processing of pri-miRNA. Hum. Mol. Genet..

[58] Livak K.J., Schmittgen T.D. (2001). Analysis of relative gene expression data using real-time quantitative PCR and the 2^−ΔΔC^_T_ Method. Methods.

[59] Morales-Pison S., Jara L., Carrasco V., Gutiérrez-Vera C., Reyes J.M., Gonzalez-Hormazabal P., Carreño L.J., Tapia J.C., Contreras H.R. (2021). Genetic Variation in MicroRNA-423 Promotes Proliferation, Migration, Invasion, and Chemoresistance in Breast Cancer Cells. Int. J. Mol. Sci..

