# Supplementary material for: A Single Variant in Pri-miRNA-155 Associated with Susceptibility to Hereditary Breast Cancer Promotes Aggressiveness in Breast Cancer Cells
Source: Int J Mol Sci. 2022 Dec 6;23(23):15418. doi: 10.3390/ijms232315418 (PMC9735695; doi:10.3390/ijms232315418)
Supplement: Supplementary file 1 [file ijms-23-15418-s001.zip › ijms-2007633-supplementary.pdf]

Supplementary Table S1. Clinical data of patients

Case	Breast cancer cases in the family	Bilateral breast cancer in the family	Ovarian cancer in the family	Age of diagnosis of the proband	Histological type
**F1**	**4**			42	Ductal
**F2**	**3**			49	Ductal
**F3**	**3**			50	Ductal
**F4**	**2**			23	Ductal In Situ
**F5**	**3**			57	Ductal In Situ
**F6**	**2**			20	Ductal In Situ
**F7**	**2**	**1**	**1**	48	Ductal In Situ
**F8**	**4**			69	Ductal In Situ
**F9**	**4**			47	Ductal In Situ
**F10**	**3**			58	Ductal In Situ
**F11**	**3**	**1**		59	Ductal In Situ
**F12**	**3**			68	Ductal In Situ
**F13**	**3**			38	Ductal Infiltrating
**F14**	**3**		**1**	49	Ductal Infiltrating
**F15**	**3**			38	Ductal Infiltrating
**F16**	**3**			55	Ductal Infiltrating
**F17**	**1**		**1**	30	Ductal Infiltrating
**F18**	**3**	**1**	**1**	40	Ductal Infiltrating
**F19**	**2**	**1**		36	Ductal Infiltrating
**F20**	**2**	**1**		32	Ductal Infiltrating
**F21**	**3**			65	Ductal Infiltrating
**F22**	**3**			45	Ductal Infiltrating
**F23**	**3**			26	Ductal Infiltrating
**F24**	**1**			29	Ductal Infiltrating
**F25**	**2**		**1**	48	Ductal Infiltrating
**F26**	**2**	**1**		30	Ductal Infiltrating
**F27**	**5**	**2**		45	Ductal Infiltrating
**F28**	**3**	**2**		37	Ductal Infiltrating
**F29**	**2**		**1**	42	Ductal Infiltrating
**F30**	**3**			46	Ductal Infiltrating
**F31**	**3**			59	Ductal Infiltrating
**F32**	**5**			55	Ductal Infiltrating
**F33**	**2**	**1**		42	Ductal Infiltrating
**F34**	**4**			46	Ductal Infiltrating
**F35**	**3**			48	Ductal Infiltrating
**F36**	**13**			65	Ductal Infiltrating
**F37**	**5**	**1**		55	Ductal Infiltrating
**F38**	**3**	**2**		34	Ductal Infiltrating
**F39**	**4**			30	Ductal Infiltrating

**F40**	**4**	**1**	**1**	37	Ductal Infiltrating
**F41**	**2**			21	Ductal Infiltrating
**F42**	**4**			50	Ductal Infiltrating
**F43**	**1**			29	Ductal Infiltrating
**F44**	**4**	**2**		32	Ductal Infiltrating
**F45**	**3**			56	Ductal Infiltrating
**F46**	**5**			54	Ductal Infiltrating
**F47**	**3**			30	Ductal Infiltrating
**F48**	**2**	**1**	**1**	48	Ductal Infiltrating
**F49**	**4**			55	Ductal Infiltrating
**F50**	**2**		**4**	58	Ductal Infiltrating
**F51**	**5**			42	Ductal Infiltrating
**F52**	**4**			58	Ductal Infiltrating
**F53**	**2**	**1**		30	Ductal Infiltrating
**F54**	**3**			54	Ductal Infiltrating
**F55**	**2**			28	Ductal Infiltrating
**F56**	**2**	**1**		34	Ductal Infiltrating
**F57**	**3**		**1**	46	Ductal Infiltrating
**F58**	**3**	**1**		42	Ductal Infiltrating
**F59**	**4**			46	Ductal Infiltrating
**F60**	**5**		**1**	53	Ductal Infiltrating
**F61**	**3**			24	Ductal Infiltrating
**F62**	**3**			36	Ductal Infiltrating
**F63**	**4**			47	Ductal Infiltrating
**F64**	**7**		**1**	59	Ductal Infiltrating
**F65**	**6**			49	Ductal Infiltrating
**F66**	**3**	**1**		58	Ductal Infiltrating
**F67**	**1**			23	Ductal Infiltrating
**F68**	**1**			28	Ductal Infiltrating
**F69**	**3**			42	Ductal Infiltrating
**F70**	**3**	**3**		35	Ductal Infiltrating
**F71**	**3**			60	In Situ
**F72**	**4**			27	In Situ
**F73**	**2**	**1**		38	In Situ
**F74**	**3**			70	In Situ
**F75**	**7**			47	In Situ
**F76**	**2**			48	Infiltrating
**F77**	**3**			46	Lobulillar
**F78**	**3**	**1**		56	Lobulillar
**F79**	**5**	**1**		53	Lobulillar In Situ
**F80**	**7**	**2**		45	Lobulillar In Situ
**F81**	**3**			74	Lobulillar Infiltrating
**F82**	**5**			47	Lobulillar Infiltrating

**F83**	**4**			49	Lobulillar Infiltrating
**F84**	**5**			44	Lobulillar Infiltrating
**F85**	**5**			46	Lobulillar Infiltrating
**F86**	**9**			50	Lobulillar Infiltrating
**F87**	**1**		**1**	31	Mixed
**F88**	**3**			32	Mucinous Infiltrating
**F89**	**3**			42	N.I.
**F90**	**5**			47	N.I.
**F91**	**3**			31	N.I.
**F92**	**3**			52	N.I.
**F93**	**4**			32	N.I.
**F94**	**4**			70	N.I.
**F95**	**4**			45	N.I.
**F96**	**3**			72	N.I.
**F97**	**4**			55	N.I.
**F98**	**3**		**1**	45	N.I.
**F99**	**4**	**1**		62	N.I.
**F100**	**5**			64	N.I.

N.I.: not identified.

Supplementary Table S2. Allele frequencies of variants found in BC patients

miRNAs	SNP	Allele frequency in BC patients (allele)		MAF[a]	*p* value[b]
**miR-10b**	rs1867863 G>T	0.570 (G)	0.430 (T)	0.4660	0.31860
	rs138423463 A>G	0.980 (A)	0.020 (G)	0.0020	0.00100
	n.300 G>T	0.995 (G)	0.005 (T)	ND	
	n.310 C>T	0.995 (C)	0.005 (T)	ND	
**miR-21**	rs570199250	0.995 (-)	0.005 (T)	0.0003	0.08300
**miR-125a**	rs12976445 C>T	0.385 (C)	0.615 (T)	0.3470	0.00001
	rs372615282 T>C	0.995 (T)	0.005 (C)	0.0001	0.04200
	rs78758318 G>A	0.985 (G)	0.015 (A)	0.0050	0.07000
	rs11881781 G>C	0.895 (G)	0.105 (C)	0.1280	0.39600
**miR-155**	rs190708267 C>T	0.990 (C)	0.010 (T)	0.0010	0.01200
**miR-195**	rs41283391 G>A	0.955 (G)	0.045 (A)	0.0410	0.71730
**miR-221**	rs191213444 G>T	0.995 (G)	0.005 (T)	0.0010	0.17300
**miR-335**	rs201306521	0.990 (-)	0.010 (T)	ND	
	rs3807348 G>A	0.495 (G)	0.485 (A)	0.5390	0.22400
	rs376491654 T>C	0.995 (T)	0.005 (C)	0.0003	0.04100
	rs41272366 T>A	0.980 (T)	0.020 (A)	0.0110	0.29100
**miR-497**	rs755634302	0.995 (-)	0.005 (T)	0.0000	0.01400

(-) without insertion; MAF: Minor allele frequency (reference allele)
[a]MAF reported in the dbSNPs database
[b]Fisher's exact test

Supplementary Table S3. List of primers

Gene	pre-miRNA (pb)	Primer sequence 5′-3′	Ta (°C)	Amplicon (bp)
**miR-10b**	110	F: TGGCAGAAGAATGAGGGAAC<br>R: ATCCATGCAACCAATTAGGC	60	532
**miR-21**	72	F: CCAGTTTTCTTGCCGTTCTG<br>R: TTTCAAAACCCACAATGCAG	60	596
**miR-125a**	86	F: TGCTGTGTCTCTGTGGCTTC<br>R: GAGGCGCTCAGAGTAGGTTG	60	600
**miR-145**	88	F: AATACACATGAGCCGTGCAG<br>R: GTGCTGAAGTTCCCACATCC	60	530
**miR-155**	65	F: TGAGCTCCTTCCTTTCAACAG<br>R: GGTTGAACATCCCAGTGACC	58	421
**miR-195/miR-497**	87	F: TGTTTGCCTTTTAAGGCTTCTC<br>R: TCTCAGCTTCGTGCTGTCTG	60	597
**miR-221**	110	F: TCCATCCAGCTTTTTCATCTC<br>R: AGACCATTTGGGTGAAATCG	60	516
**miR-222**	112	F: TTTGTTTGCTGCTGGATCTC<br>R: TGCCCAATAATCTCTCTCAGG	60	374
**miR-335**	94	F: CCACCATTGGTTCTCTCTCC<br>R: TTTTGTTCTTGAGAATCTGTGC	58	550
**miR-520c**	87	F: TGCAGATGGTCCTTTTAGGG<br>R: CAGCAAGAACGAAACTCCATC	60	372

Ta: annealing temperature
