# Supplementary figures and images for: A Single Variant in Pri-miRNA-155 Associated with Susceptibility to Hereditary Breast Cancer Promotes Aggressiveness in Breast Cancer Cells
Source: Int J Mol Sci. 2022 Dec 6;23(23):15418. doi: 10.3390/ijms232315418 (PMC9735695; doi:10.3390/ijms232315418)

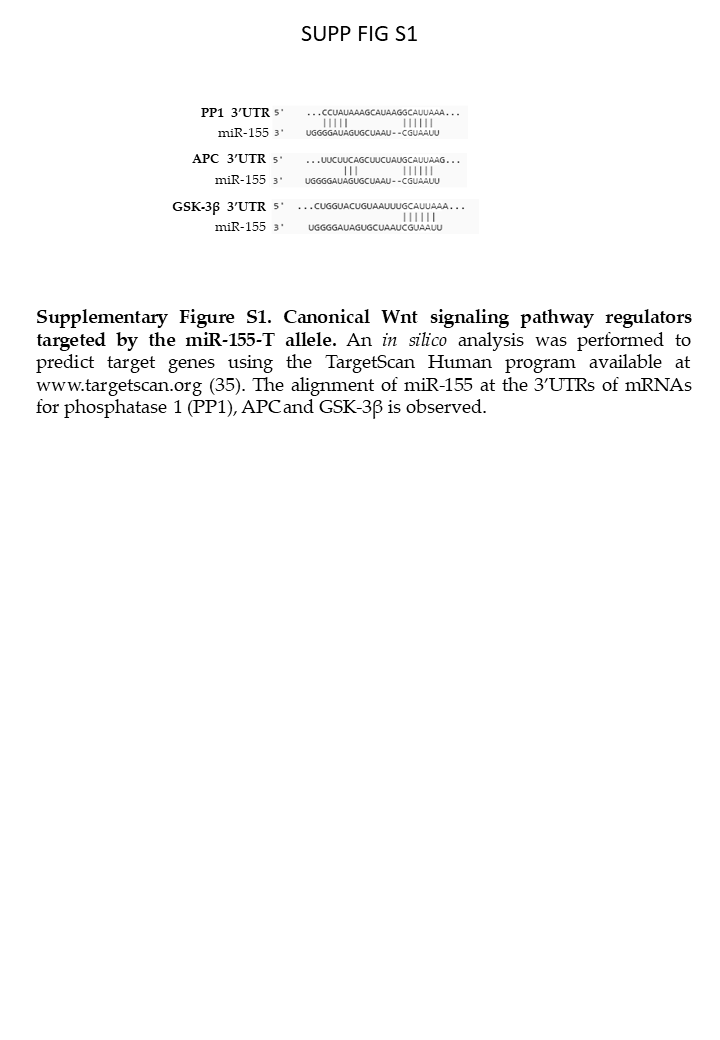

Supplement: Supplementary file 1 [file ijms-23-15418-s001.zip › fig S1.TIF]

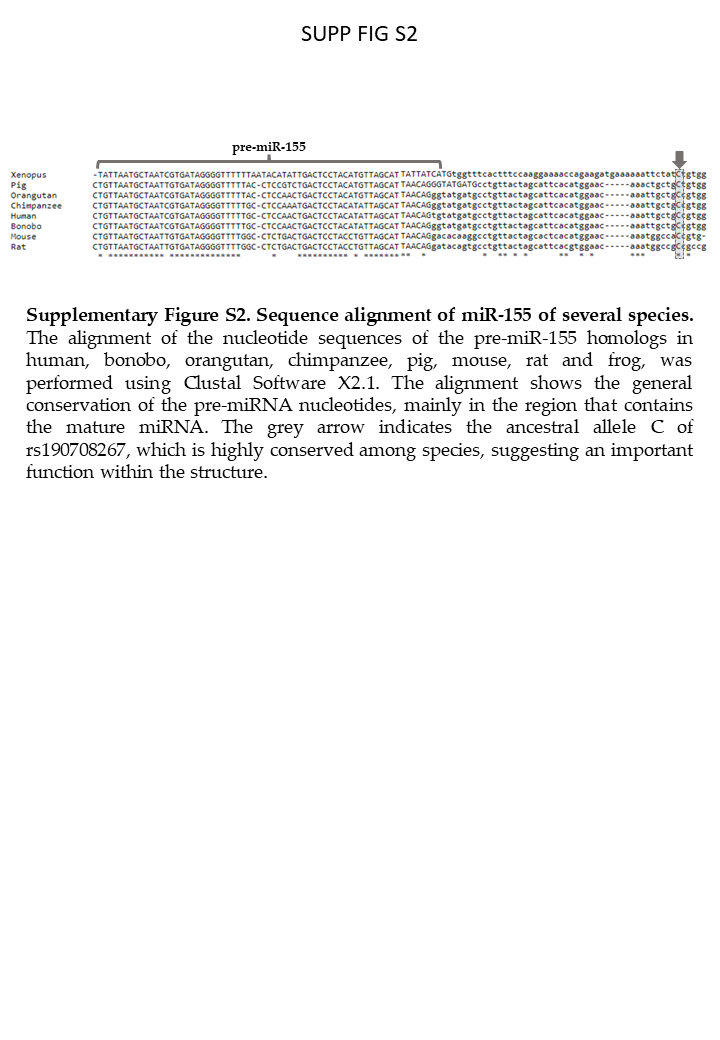

Supplement: Supplementary file 1 [file ijms-23-15418-s001.zip › fig S2.TIF]

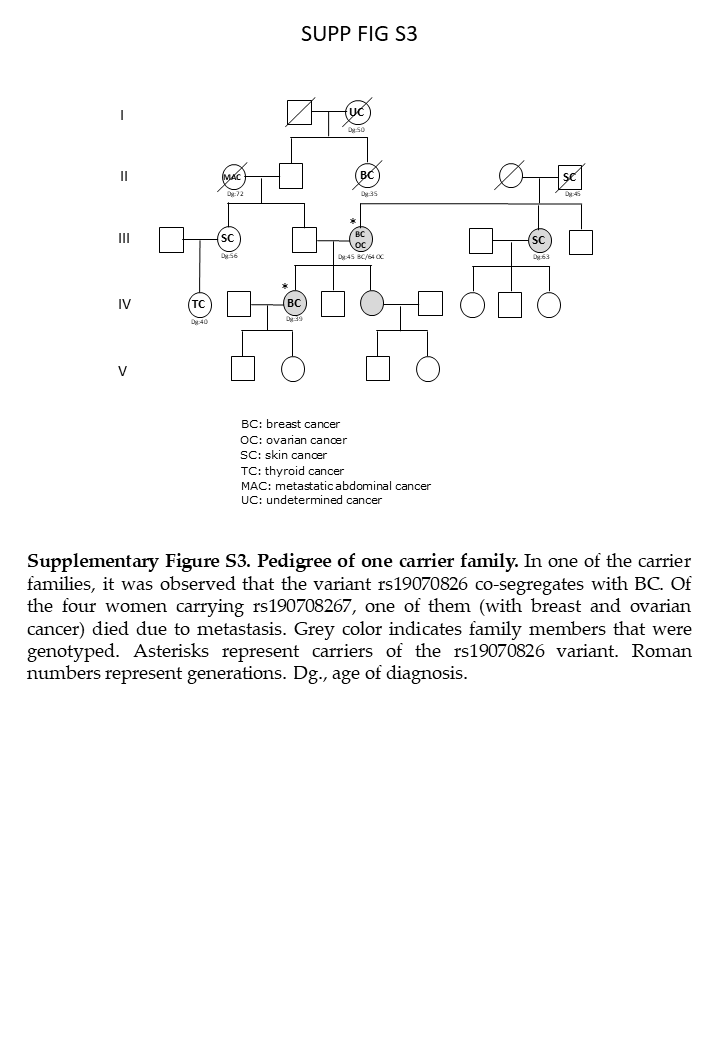

Supplement: Supplementary file 1 [file ijms-23-15418-s001.zip › fig S3.TIF]
